# Effect of the Consolidation Level on Organic Volatile Compound Emissions from Maize during Storage

**DOI:** 10.3390/ma16083066

**Published:** 2023-04-13

**Authors:** Aleksandra Żytek, Robert Rusinek, Anna Oniszczuk, Marek Gancarz

**Affiliations:** 1 Institute of Agrophysics Polish Academy of Sciences, Doświadczalna 4, 20-290 Lublin, Poland; 2 Department of Inorganic Chemistry, Medical University of Lublin, Chodźki 4a, 20-093 Lublin, Poland; 3 Faculty of Production and Power Engineering, University of Agriculture in Krakow, Balicka 116B, 30-149 Krakow, Poland

**Keywords:** maize, consolidation, storage, electronic nose, GC-MS, chemometrics

## Abstract

The aim of this study was to determine the emission of organic volatile compounds from maize grain as a function of granularity and packing density of bulk material in conditions imitating processes occurring in silos. The study was carried out with the use of a gas chromatograph and an electronic nose, which was designed and constructed at the Institute of Agrophysics of PAS and has a matrix of eight MOS (metal oxide semiconductor) sensors. A 20-L volume of maize grain was consolidated in the INSTRON testing machine with pressures of 40 and 80 kPa. The control samples were not compacted, and the maize bed had bulk density. The analyses were carried out at a moisture content of 14% and 17% (w.b.—wet basis). The measurement system facilitated quantitative and qualitative analyses of volatile organic compounds and the intensity of their emission during 30-day storage. The study determined the profile of volatile compounds as a function of storage time and the grain bed consolidation level. The research results indicated the degree of grain degradation induced by the storage time. The highest emission of volatile compounds was recorded on the first four days, which indicated a dynamic nature of maize quality degradation. This was confirmed by the measurements performed with electrochemical sensors. In turn, the intensity of the volatile compound emission decreased in the next stage of the experiments, which showed a decline in the quality degradation dynamics. The sensor responses to the emission intensity decreased significantly at this stage. The electronic nose data on the emission of VOCs (volatile organic compounds) as well as grain moisture and bulk volume can be helpful for the determination of the quality of stored material and its suitability for consumption.

## 1. Introduction

Maize (*Zea mays* L.) is one of the most important sources of feed and food plant material cultivated worldwide. In 2020, the global leaders in the production of this plant were the USA (approx. 360.25 million tons) and China (260.67 million tons). The production in the European Union amounted to 67.84 million tons with 30.29 million tons accounting for approximately 15% of the global maize trade provided by the largest European producer of this cereal, i.e., Ukraine [1]. The Russian invasion of Ukraine in 2022 disrupted agricultural exports from the region and raised uncertainty about supplies, simultaneously driving up commodity prices and increasing market instability. High demands, limitations in supplying agricultural products, and long-term drought are known to lead to an additional increase in cereal and food commodity prices [2].

Cereals, including maize, are an important source of carbohydrates and protein, but large amounts of grain affected by fungal diseases and exposed to the presence of pests are lost. Poor grain storage conditions may support fungal flora growth in the plant material. The most common pathogens of maize grains are fungi of the genera *Fusarium*, *Alternaria*, *Aspergillus*, and *Penicillium*, which may pose a health threat when consumed by humans and animals [3]. The presence of pathogens in food products has prompted many countries to establish maximum allowable levels of mycotoxins in food in order to prevent their adverse effects on the health of consumers [4]. *Aspergillus* fungi can infect plants during their growth, and harvested grains may contain aflatoxins whose concentration may increase during storage. Their presence in agricultural products is unavoidable, although the contamination level can be reduced by controlling the growth of these pathogens. The temperature, humidity, and O_2_ and CO_2_ concentrations should particularly be monitored. The optimal moisture content in maize grains is 14% (w.b.), whereas a moisture content exceeding 16% (w.b.) poses a high risk of grain damage and infection [5].

Harvested grain is often stored in silos to ensure appropriate conditions and prevent degradation of the material. Silos are engineered structures used for storage, processing, and distribution of bulk materials. They are most often used in industry and agriculture [6]. Elevated temperature and higher humidity in grain silos can lead to losses of stored grain and a decline in its nutritional value [7,8]. Elimination of gas exchange and maintenance of the initial moisture content can reduce the deterioration of the quality of grain stored in silos. Hermetic storage ensures stable thermal conditions and the longevity of grain [9]. Differences in the silo temperature caused by bed heating may increase the moisture content and aggravate the potential losses of the raw material. As reported by Gawrysiak-Witulska et al., 2016, storage humidity, temperature, and pressure exert an impact on the content of bioactive compounds [10]. The friction coefficient and the grain bed size have an influence on the pressure exerted by the grain stored in the silo. The density of consolidated material increases with increasing moisture due to the higher grain deformation [11,12]. The physical and mechanical properties of maize grain have been studied by many researchers [13,14,15,16]. Often, low molecular weight compounds initially predominate in storage but may eventually give way to a larger number of higher molecular weight compounds. This shift may be due to the growth of fungi/yeast etc. and then transition to secondary metabolism—which may result due to oxygen depletion. Similar research results were obtained by the authors in their previous research on the storage of rape seeds [17,18].

There are many instrumental and laboratory techniques for monitoring the quality of stored agricultural materials. Laboratory methods involving the determination of fungal microflora or the content of ergosterol, which is an indicator of the presence of fungal biomass in stored materials, are time-consuming and cost-inefficient approaches. Given the recent development of techniques for the analysis of volatile organic compounds, these methods can be used for the assessment of the biodegradation status of agricultural materials. In addition to precise chromatographic techniques, the electronic nose is often used in this type of research. Analyses performed with the use of this versatile device can determine, e.g., the intensity of the emission of volatile organic compounds, which is a biodegradation marker. There are many literature data showing a positive correlation between high emission intensity and high dynamics of negative microbiological changes [19,20,21].

The aim of this study was to analyze the emission of volatile organic compounds as a function of maize bed consolidation and moisture. This study was conducted with the use of an electronic nose consisting of eight MOS sensors and with a gas chromatograph. Maize grain with a volume of 20 L was compacted in the INSTRON testing machine with consolidation pressures of 40 and 80 kPa. The measurement system facilitated the analysis of volatile organic compounds and emission intensity during 30-day storage. The analyses revealed the volatile organic compound profile as a function of storage time and the degree of bed consolidation and moisture (w.b.).

## 2. Materials and Methods

### 2.1. Materials

The medium-early Opoka—FAO 240 maize variety was used in this study. The material was obtained from Nowosiółki in Telatyn commune, Poland, and cultivated on black earth. The maize grains used in this study were unconsolidated (bulk density) and consolidated under the pressures of 40 and 80 kPa, which corresponds to the pressure noted in industrial silos. Similar research results were obtained by the authors in their previous research on the storage of rape seeds [22]. The bulk density in silos was analyzed in other studies as well [23]. The Instron 8872 testing machine was used to consolidate the material in cylinders equipped with VOC intake valves [24]. The maize grains had standard 14% (w.b.) and elevated 17% moisture content (w.b.). To accelerate fungal flora growth in the analyzed material, the maize-containing cylinders were placed in silos with an elevated temperature of 32 °C. The analyses were performed during 30-day storage of the material. This paper presents only the results from the first nine storage days, when changes in the qualitative degradation were observed. During the following 21 days, the material exhibited no significant changes in the emission of volatile organic compounds. Since storage day 9, there were no quantitative and qualitative changes in the VOC emission; therefore, these results are not presented.

### 2.2. Electronic Nose

An Agrinose electronic nose constructed at the Institute of Agrophysics, Polish Academy of Sciences (Lublin, Poland) was used in the analyses (Table S3. Supplementary Files) [25,26,27]. It consists of eight MOS gas sensors (AS–MLV-P2—CO, butane, methane, ethanol, and hydrogen; specifically designed for volatile organic compounds; TGS2602—ammonia, hydrogen sulfide, high sensitivity to VOCs, and odorous gases; TGS2603—odors generated from spoiled foods; TGS2612—methane, propane, and butane; TGS2610—LP gas and butane; TGS2611—natural gas and methane; TGS2620—solvent vapors, volatile vapors, and alcohol; TGS2600- general air contaminants, hydrogen, and carbon monoxide). The sensor response comprises three parameters: maximum sensor response ∆R/R_max_, which indicates the intensity of VOC emission; response time t_IM_; and the time of removal of the substance from the sensor t_CL0_. Similar methods have previously been used to detect fungal infections in cereal grains, including maize [21,28]. In these studies, only the maximum sensor response∆R/R_max_ to the intensity of VOC emission was determined with the use of the electronic nose.

### 2.3. GC–MS Analysis

The intensity of the signals of volatile organic compounds contained in the maize grains was determined with the use of a Trace GC Ultra gas chromatograph (ThermoFisher Scientific, Waltham, MA, USA) integrated with an ITQ 1100 mass spectrometer (ThermoFisher Scientific, Waltham, MA, USA). An SPME (solid-phase micro-extraction) fiber with an absorbent (50/30 μm Divinylbenzene/Carboxene/Polydimethylsiloxane (DVB/CAR/PDMS), Stableflex (2 cm) 24 Ga (Sigma Aldrich, Poznań, Poland)) was placed in the measuring chamber for 30 min together with the material emitting volatile organic compounds to adsorb the compounds on the fiber surface. Next, it was transferred to a GC injector for 5 min to desorb the VOCs. A Zebron ZB-5Msplus Capillary GC 30 m × 0.25 mm × 0.25 um column was used for the analysis. The injection temperature was 60 °C for 5 min and then increased from 60 to 250 °C at a rate of 5 °C/min and from 250 to 270 °C at 10 °C/min. The final temperature was held for 5 min. The helium flow rate was kept constant at 2.2 mL/min. The Wiley 138 library was used to identify the compounds with greater than 80% matches. Only one compound had a Wiley 138 library match of less than 80%. Therefore, for this one compound, based on the literature data and with a low level of match of the other identified compounds for this retention time, it was considered that these are sufficient grounds to include this one compound in the presented study results, which has a lower level of match but over 60%. The obtained results were compared with the literature data for similar materials [29].

### 2.4. Statistical Analysis

The statistical analysis (variance, principal component analysis, and simple correlations) was performed at the significance level α = 0.05 using TIBCO Statistica software (version 12.0, StatSoft Inc., Palo Alto, CA, USA). Maize grains subjected to the different pressure values and with the different moisture levels were analyzed. The principal components were correlated with the sensor response ΔR/R_max_ and the T_ratio_ for the eight sensors used to determine the volatile organic compounds emitted by the maize grains. The optimal number of principal components was determined based on the Cattel criterion. To evaluate the ability of the Agrinose to identify the maize odor profile using principal component analysis (PCA), a data matrix of 18 columns (sensor responses and groups of compounds) and 9 rows (experimental days) was constructed. The input matrix was scaled automatically.

## 3. Results and Discussion

### 3.1. Electronic Nose Responses

The results of the comparative analysis performed using the Agrinose device corresponded to the results of the chemometric analysis. The values of the e-nose sensor response decreased with the storage time and the progressive degradation of the material (Supplementary Files—Table S1). The storage experiment can be divided into two stages. The first four days were characterized by a dynamic degradation process reflected in increased VOC emissions, whereas stabilization and deceleration of the process were recorded on the successive days. Similar results were reported in an experiment with rapeseed [17]. The results of the analysis of the sensor responses to the volatile organic compounds present in the maize grains were correlated with the GC–MS analysis results. Similar studies have been conducted on volatile source analyses in maize grain [8,30].

### 3.2. Identification of Major Volatile Organic Compounds—GC–MS Analysis

The analysis of the volatile organic compounds contained in the maize kernels revealed the presence of 69 different chemical substances constituting the odor profile in this stored material. These compounds were assigned to nine different groups: alcohols, acids, ketones, esters, hydrocarbons, azines, terpenes, aldehydes, and others (Supplementary Files—Table S2). 4-(benzoyloxy)-2H-pyran-3-one, representing the group of ketones, was the dominant compound detected in most samples. In general, the profile of volatile organic compounds varied depending on the consolidation pressure and the grain bed moisture. The detected chemicals were identified with the use of the Wiley library. Similar spectrometric analyses of other cereals and their products were performed previously [31,32,33,34].

### 3.3. Principal Component Analysis (PCA)

**Figure 1:**
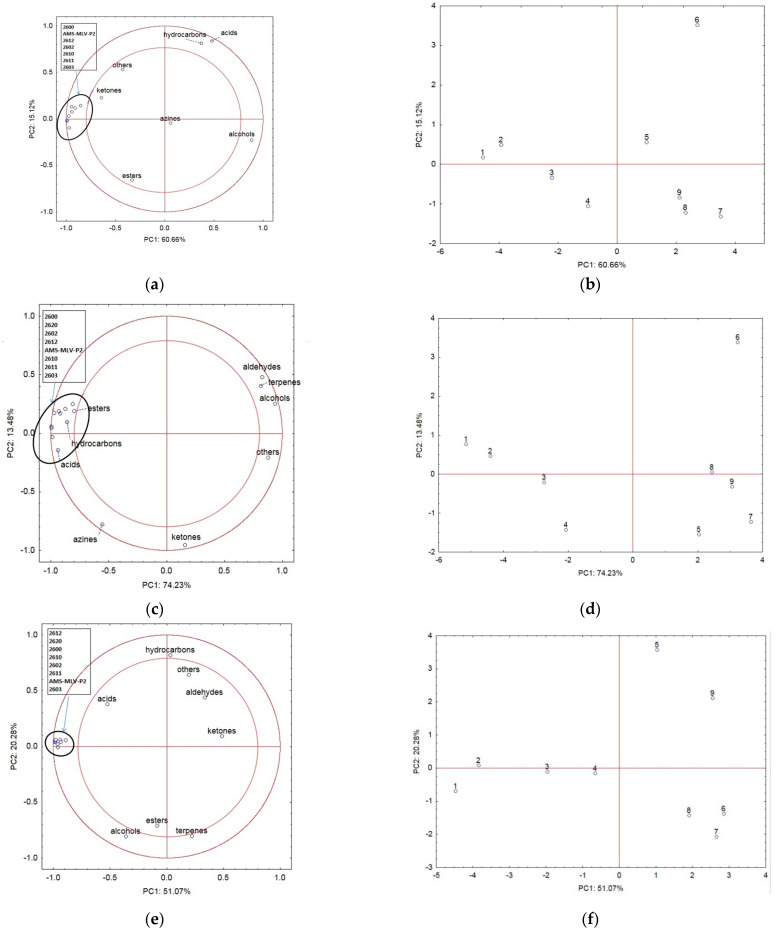

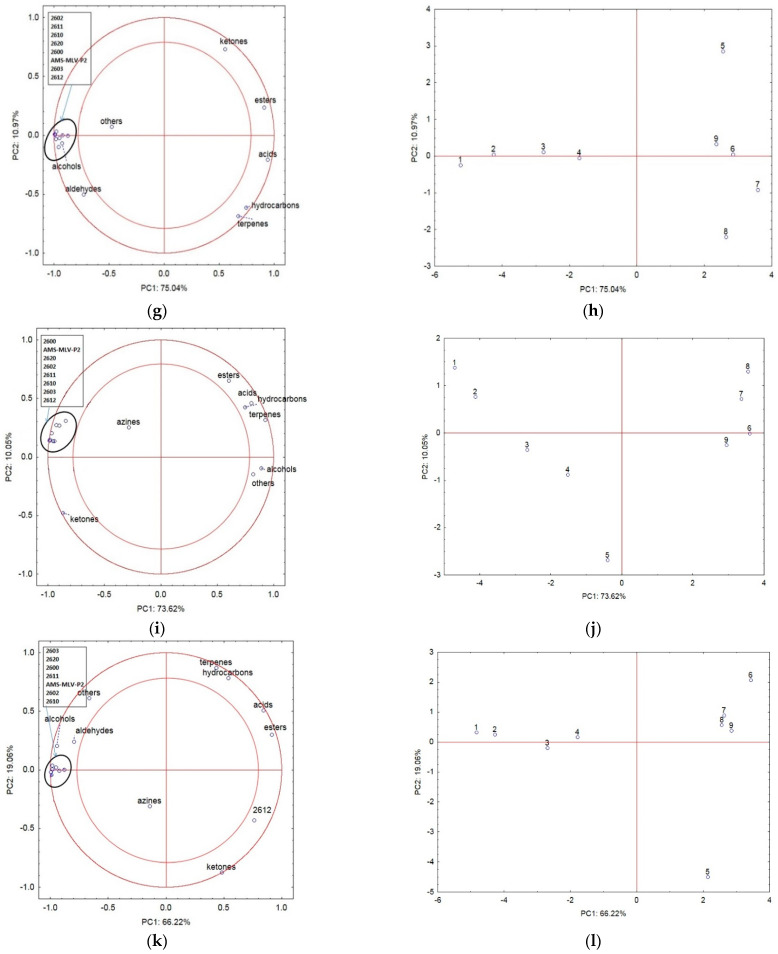
Loading plot (**a**) and score plot (**b**) of the principal component analysis carried out for chemical groups and sensor responses to changes in VOCs for nine days of corn grain storage at 14% (w.b.) moisture and 0 kPa consolidation. Loading plot (**c**) and score plot (**d**) of the principal component analysis carried out for chemical groups and sensor responses to changes in VOCs for nine days of corn grain storage at 17% (w.b.) moisture and 0 kPa consolidation. Loading plot (**e**) and score plot (**f**) of the principal component analysis carried out for chemical groups and sensor responses to changes in VOCs for nine days of corn grain storage at 14% (w.b.) moisture and 40 kPa consolidation. Loading plot (**g**) and score plot (**h**) of the principal component analysis carried out for chemical groups and sensor responses to changes in VOCs for nine days of corn grain storage at 17% (w.b.) moisture and 40 kPa consolidation. Loading plot (**i**) and score plot (**j**) of the principal component analysis carried out for chemical groups and sensor responses to changes in VOCs for nine days of corn grain storage at 14% (w.b.) moisture and 80 kPa consolidation. Loading plot (**k**) and score plot (**l**) of the principal component analysis carried out for chemical groups and sensor responses to changes in VOCs for nine days of corn grain storage at 17% (w.b.) moisture and 80 kPa consolidation.

Figure 1a,c,e,g–i,k illustrates the projection of the variables in the different variants of consolidation and moisture of the maize grains. The principal components explain the correlations between the compounds identified in the analysis and the MOS responses. Figure 1b,d,f,h,j,l shows the changes observed in the VOC emissions correlated with the responses of the e-nose sensors during storage.

Figure 1a shows the projection of the variables of the samples with 14% (w.b.) moisture and 0 kPa consolidation on the PC1 (60.66%) and PC2 (15.12%) planes. The first two principal components PC1 and PC2 explain 75.78% of the variability of the system. There was a strong negative correlation between the MOS responses associated with the VOC emission intensity and the changes in the proportion of alcohols in the detected volatile substances. The first principal component, describing PC1 in over 60%, explains the correlations between the changes in the content of ketones and alcohols and the sensor responses. The increasing alcohol content was found to decrease the MOS responses. The sensor responses were positively correlated with the presence of ketones. Additionally, esters exhibited a strong negative correlation with acids and hydrocarbons. The group of azines exerted no influence on the variability of the system. The first principal component PC1 in Figure 1b shows the degree of the progressive qualitative degradation of the maize grains during nine storage days. The axis of the first principal component PC1 divides the grain storage period into two stages: the first days of storage are located on the negative side (rapid progression of the spoilage process), and the successive days are located on the positive side of PC1 (slow progression of the degradation process). The MOS responses on the negative side of PC1 (storage days 1–4) exhibit a strong positive correlation with the emission of ketones and a negative correlation with the emission of alcohols. Studies by Worku et al., 2022 [35], also demonstrated the appearance of alphatoxins as well as the loss of nutrients during storage using various storage techniques.

Figure 1c shows the projection of variables of the samples with 17% (w.b.) moisture content and 0 kPa consolidation on the PC1 (74.23%) and PC2 (13.48%) planes (87.71% in total). In the samples with the higher moisture content, the detected groups of compounds exhibited a strong interaction with the MOS responses in comparison with the above-described samples with 14% moisture (w.b.). A strong positive correlation was found in the MOS responses with esters, hydrocarbons, and acids, while a negative correlation was observed with aldehydes, terpenes, and alcohols. Azines were found to be strongly negatively correlated with aldehydes, terpenes, and alcohols. Only ketones in this case did not exhibit a significant correlation with the other compounds and sensor responses to the intensity of VOC emission. The PC1 values (Figure 1d) indicate changes in VOC emissions, which were strongly correlated with the responses of the e-nose sensors during storage. The negative PC1 values reflect the first storage days, and the positive PC1 values represent the successive days of maize grain storage, as in the variant with the 14% (w.b.) moisture content and 0 kPa consolidation. Odjo, N. et al., 2022 [9], examined the effect of storage techniques on the variability of p-coumaric acid and ferulic acid, which totaled from 6.4% to 24.1% and from 6.2% to 24.3%, respectively. There were significant differences in these parameters for the samples before and after storage, even if no clear trend emerged.

As shown in Figure 1e, the groups of hydrocarbons, aldehydes, and others were negatively correlated with esters, alcohols, and terpenes. There was no significant correlation of these groups with the MOS responses. The first two principal components PC1 (51.07%) and PC2 (20.28%) explain 71.35% of the variability of the system. In turn, a strong positive relationship was observed between the sensor responses and the changes in the content of acids during the storage period. Similar to the results presented above, the first principal component describes the progress of qualitative degradation of the maize grains during storage (Figure 1f). As shown in Table S2 (Supplementary Files), a significant increase in the content of aldehydes and a decrease in hydrocarbons were recorded in the 14% (w.b.) moisture and 40 kPa consolidation variant from day 5 of the experiment. The emission of VOCs recorded as MOS responses declined during storage. In their work, Jia et al., 2022 [36], also showed that storage time and conditions can significantly affect aroma quality. They performed storage tests in low temperature and vacuum conditions, which allows for the effective preservation of the quality of the aroma.

In the 40 kPa consolidation and 17% (w.b.) moisture variant shown in Figure 1g, the first two principal components PC1 (75.04%) and PC2 (10.97%) accounted, in total, for 86.01%. Terpenes and hydrocarbons exhibited a positive correlation with each other; likewise, aldehydes and alcohols exhibited a positive correlation with each other. Esters and acids were strongly negatively correlated with the MOS responses and alcohols. The relationships presented in Figure 1h show negative values of the first principal component PC1 during the first four days of storage and positive values of PC1 on the successive storage days. The aim of the research of Usseglio et al., 2017 [37], was to assess the share of volatile organic compounds emitted by the fungus–maize system in grain–insect interactions. The VOCs emitted by fungus-infected grains were enriched with alcohols, ketones, and sesquiterpenes, which were considered early indicators of fungal infection.

As shown in Figure 1i, the groups of esters, acids, terpenes, hydrocarbons, alcohols, and others were strongly and positively correlated with each other and strongly but negatively correlated with ketones and the MOS responses. The first two components PC1 (73.62%) and PC2 (10.05%) for the samples in the 80 kPa consolidation and 14% (w.b.) moisture variant explain 83.67% of the variability of the system. The group of azines was found to exert only a slight impact on the variability of the system. In Figure 1j, the PC1 values denote the successive days of the experiment. The volatile organic compounds emitted by healthy corn grains differ from those emitted by grains infected with fungi [37].

Figure 1k shows the 80 kPa consolidation and 17% (w.b.) moisture variant in which the first two principal components PC1 (66.22%) and PC2 (19.06%) explain, in total, 85.28% of the variability of the system. The group of azines was characterized by a low correlation between the changes and the maize storage time. Alcohols and aldehydes were positively correlated with the e-nose sensor response with the exception of the sensor 2612 responses to these compounds. Acids and esters as well as terpenes and hydrocarbons were strongly negatively correlated with the MOS responses and alcohols and aldehydes. In Figure 1l, PC1 reflects the progress of qualitative degradation of the maize grains during storage. The negative PC1 values define the first four days of storage, and the PC2 values reflect the further days of storage of the material in the specified conditions. Usseglio et al., 2017 [37], assessed the share of VOCs emitted by the fungus–maize system. The volatile organic compounds emitted by fungus-infected grains emitted alcohols, ketones, and sesquiterpenes, which were considered early indicators of fungal infection.

The tests carried out prove that there is an influence between the physical parameters of the plant material and the type of volatile organic compounds and the level of emission of these compounds during storage of the raw material for which an electronic nose and a gas chromatograph coupled with a mass spectrometer were used.

## 4. Conclusions

This study determined the profile of volatile compounds for the storage time and the degree of consolidation of the tested maize grains. As shown by the present experiments and chemometric analyses, the first principal component PC1 described the progressive qualitative degradation of the maize grains during storage in all variants. On the first four days of storage, the quality deterioration process was significant, which was reflected in the higher VOC emissions. This relationship was confirmed by the measurements carried out using the MOS sensors. In turn, the intensity of the volatile organic compound emissions decreased in the next stage of the experiments, which indicated a decline in the dynamics of the quality degradation of the maize grains. The MOS responses to the emission intensity at this stage decreased significantly.

The responses of the e-nose sensors shown in the loading plot were always located on their left side and were positively correlated with alcohols for the 17% wet basis material with increased consolidation. Chemical compounds from the azine group detected in the material with 14% (w.b.) moisture had no effect on the reaction of the sensors for the tested material. The group of azines detected in the material with 14% (w.b.) moisture exerted only a slight effect on the variability of the system. The electronic nose data on the VOC emissions, moisture, and grain bulk volume can be helpful in the determination of the quality and suitability for consumption of stored materials.

## Data Availability

Not applicable.

## Supplementary Materials

The following supporting information can be downloaded at: https://www.mdpi.com/article/10.3390/ma16083066/s1. Table S1. The mean values of the obtained parameters using the Agrinose for different moisture content and consolidation for 9 days of the corn grain storage. Table S2. Percentage share of groups of volatile organic compounds for different moisture content and consolidation for 9 days of the corn grain storage. Table S3. Technical data of Agrinose sensors.
