# Supplementary material for: Effect of the Consolidation Level on Organic Volatile Compound Emissions from Maize during Storage
Source: Materials (Basel). 2023 Apr 13;16(8):3066. doi: 10.3390/ma16083066 (PMC10145107; doi:10.3390/ma16083066)
Supplement: Supplementary file 1 [file materials-16-03066-s001.zip › materials-2302869-supplementary.pdf]

Supplementary Files

Table S1. The mean values of the obtained parameters using the Agrinose for different moisture content and consolidation for 9 days of the corn grain storage.

Consolidation	Moisture content	Days	2602	AMS-MLV-P2	2603	2612	2610	2611	2620	2600
0 kPa	14%	1	0.33	0.40	0.05	0.01	0.26	0.25	0.48	0.42
		2	0.30	0.37	0.05	0.01	0.23	0.23	0.43	0.38
		3	0.21	0.24	0.04	0.01	0.20	0.19	0.26	0.19
		4	0.15	0.16	0.04	0.01	0.17	0.16	0.16	0.10
		5	0.15	0.18	0.02	0.00	0.12	0.12	0.23	0.19
		6	0.08	0.10	0.01	0.00	0.07	0.07	0.13	0.11
		7	0.05	0.08	0.01	-0.00	0.04	0.04	0.08	0.07
		8	0.11	0.14	0.01	0.00	0.09	0.10	0.17	0.14
		9	0.10	0.11	0.01	0.00	0.08	0.08	0.15	0.13
	17%	1	0.65	0.60	0.13	0.08	0.44	0.42	0.82	0.72
		2	0.59	0.54	0.12	0.07	0.40	0.38	0.75	0.66
		3	0.42	0.35	0.11	0.07	0.34	0.33	0.45	0.33
		4	0.30	0.23	0.10	0.07	0.29	0.28	0.27	0.16

		**5**	0.27	0.19	0.02	0.04	0.16	0.17	0.29	0.23
		**6**	0.25	0.16	0.01	0.05	0.13	0.13	0.24	0.20
		**7**	0.20	0.15	0.01	0.03	0.11	0.11	0.21	0.17
		**8**	0.22	0.19	0.02	0.04	0.15	0.17	0.28	0.23
		**9**	0.21	0.15	0.02	0.04	0.14	0.14	0.25	0.21
**40 kPa**	**14%**	**1**	0.34	0.41	0.06	0.03	0.28	0.28	0.52	0.46
		**2**	0.31	0.37	0.06	0.02	0.26	0.25	0.48	0.42
		**3**	0.22	0.24	0.05	0.02	0.22	0.22	0.29	0.21
		**4**	0.16	0.16	0.05	0.02	0.19	0.18	0.17	0.10
		**5**	0.14	0.17	0.02	0.01	0.12	0.12	0.22	0.18
		**6**	0.09	0.10	0.01	0.00	0.07	0.06	0.13	0.11
		**7**	0.05	0.08	0.01	0.00	0.04	0.04	0.08	0.06
		**8**	0.11	0.14	0.01	0.00	0.09	0.10	0.18	0.15
		**9**	0.10	0.10	0.01	0.01	0.08	0.08	0.15	0.13
	**17%**	**1**	0.55	0.53	0.11	0.06	0.36	0.34	0.68	0.60
		**2**	0.50	0.48	0.10	0.06	0.32	0.31	0.62	0.54
		**3**	0.36	0.31	0.10	0.05	0.28	0.26	0.37	0.27

		**4**	0.26	0.20	0.09	0.05	0.23	0.22	0.22	0.14
		**5**	0.14	0.14	0.02	0.01	0.11	0.12	0.19	0.16
		**6**	0.09	0.10	0.01	0.03	0.08	0.08	0.14	0.11
		**7**	0.06	0.09	0.01	0.01	0.06	0.07	0.11	0.09
		**8**	0.10	0.14	0.02	0.03	0.10	0.12	0.18	0.15
		**9**	0.08	0.10	0.01	0.02	0.09	0.10	0.15	0.12
**80 kPa**	**14%**	**1**	0.53	0.51	0.09	0.05	0.36	0.34	0.68	0.60
		**2**	0.48	0.46	0.08	0.04	0.33	0.31	0.62	0.55
		**3**	0.34	0.30	0.08	0.04	0.28	0.27	0.37	0.27
		**4**	0.25	0.19	0.08	0.04	0.24	0.23	0.22	0.14
		**5**	0.21	0.22	0.02	0.01	0.16	0.16	0.31	0.26
		**6**	0.10	0.11	0.01	0.01	0.08	0.08	0.16	0.13
		**7**	0.10	0.12	0.01	0.00	0.08	0.08	0.16	0.14
		**8**	0.09	0.13	0.01	0.00	0.10	0.10	0.17	0.14
		**9**	0.09	0.09	0.01	0.01	0.09	0.09	0.16	0.14
	**17%**	**1**	0.48	0.49	0.09	0.00	0.31	0.30	0.59	0.52
		**2**	0.43	0.45	0.09	0.00	0.29	0.27	0.54	0.47

		**3**	0.31	0.29	0.08	0.00	0.24	0.23	0.32	0.24
		**4**	0.22	0.19	0.08	0.00	0.21	0.20	0.19	0.12
		**5**	0.10	0.12	0.01	0.01	0.10	0.11	0.17	0.13
		**6**	0.06	0.09	0.01	0.01	0.06	0.07	0.11	0.09
		**7**	0.04	0.08	0.01	0.00	0.05	0.06	0.09	0.07
		**8**	0.06	0.11	0.01	0.01	0.08	0.10	0.13	0.11
		**9**	0.04	0.08	0.01	0.00	0.07	0.08	0.11	0.09

Table S2. Percentage share of groups of volatile organic compounds for different moisture content and consolidation for 9 days of the corn grain storage.

Consolidation	Moisture content	Days	Alcohols	Acids	Ketones	Esters	Hydrocarbons	Azines	Terpenes	Aldehydes	Others
0 kPa	14%	1	12.50	1.44	4.33	75.96	1.44	1.92	n.d.	n.d.	2.40
		2	11.90	1.64	4.42	76.16	1.54	1.91	n.d.	n.d.	2.42
		3	12.10	1.74	4.22	76.06	1.24	1.90	n.d.	n.d.	2.73
		4	13.43	1.93	4.68	84.43	1.38	2.11	n.d.	n.d.	3.03
		5	14.42	2.33	4.19	73.95	1.86	1.86	n.d.	n.d.	1.40
		6	13.77	5.26	3.64	70.04	2.02	2.02	n.d.	n.d.	3.24

		7	14.56	1.94	2.43	76.21	1.46	1.94	n.d.	n.d.	1.46
		8	14.78	1.97	1.97	75.86	1.48	2.46	n.d.	n.d.	1.48
		9	15.17	1.90	4.27	74.41	1.42	1.42	n.d.	n.d.	1.42
	17%	1	n.d.	22.91	37.44	18.06	18.50	3.08	n.d.	n.d.	n.d.
		2	0.39	21.07	38.35	18.40	18.74	3.04	n.d.	n.d.	n.d.
		3	0.61	20.49	37.95	18.11	19.32	3.03	n.d.	n.d.	0.50
		4	0.68	22.74	42.12	20.10	21.44	3.36	n.d.	n.d.	0.56
		5	5.66	15.09	43.94	15.09	7.01	2.70	n.d.	3.50	7.01
		6	13.22	10.34	31.90	16.95	12.64	n.d.	4.60	6.61	3.74
		7	11.21	8.01	44.62	12.81	9.61	2.52	3.66	2.52	5.03
		8	11.82	13.64	38.86	15.00	7.73	2.73	4.09	2.95	3.18
		9	12.56	11.88	39.91	13.45	8.74	2.47	3.36	2.69	4.93
40 kPa	14%	1	20.65	11.59	28.97	11.84	19.40	n.d.	4.28	n.d.	3.27
		2	19.85	12.79	27.97	11.54	20.95	n.d.	3.33	n.d.	3.57
		3	20.25	12.87	27.97	11.46	20.66	n.d.	3.21	n.d.	3.57
		4	22.48	14.28	31.04	12.72	22.93	n.d.	3.56	n.d.	3.97
		5	10.82	15.08	32.79	10.82	25.25	n.d.	n.d.	n.d.	5.25

		6	19.08	8.92	35.38	12.31	14.77	n.d.	5.23	n.d.	4.31
		7	21.44	10.33	27.29	13.84	19.49	n.d.	4.68	n.d.	2.92
		8	17.39	9.21	32.74	13.30	18.16	n.d.	5.63	n.d.	3.58
		9	15.07	7.86	29.48	6.33	28.38	n.d.	4.37	5.02	3.49
	17%	1	41.70	6.56	18.53	10.42	2.32	n.d.	3.09	11.20	6.18
		2	40.70	7.56	19.53	11.42	2.42	n.d.	3.09	9.20	6.08
		3	41.88	7.37	18.53	12.25	2.12	n.d.	3.08	8.76	6.01
		4	46.49	8.18	20.57	13.59	2.35	n.d.	3.42	9.72	6.67
		5	10.64	9.84	48.40	19.95	2.66	n.d.	3.19	2.39	2.93
		6	15.17	10.20	24.88	21.39	14.43	n.d.	4.73	2.74	6.47
		7	11.84	12.34	25.19	20.65	14.11	n.d.	5.04	7.81	3.02
		8	13.23	10.90	23.20	15.31	20.65	n.d.	6.50	7.42	2.78
		9	11.24	9.88	26.94	22.09	11.43	n.d.	3.88	6.98	7.56
80 kPa	14%	1	n.d.	6.22	64.73	21.58	2.07	2.49	n.d.	n.d.	2.90
		2	1.01	5.24	65.63	20.57	2.07	2.53	n.d.	n.d.	2.95
		3	2.11	5.04	65.33	20.46	2.09	2.33	n.d.	n.d.	2.63
		4	2.34	5.60	72.52	22.71	2.33	2.58	n.d.	n.d.	2.92

		5	4.04	3.68	70.96	15.07	2.57	n.d.	n.d.	n.d.	3.68
		6	10.85	8.94	35.53	28.30	7.45	n.d.	5.11	n.d.	3.83
		7	5.67	13.15	34.69	26.08	7.94	3.17	5.22	n.d.	4.08
		8	5.53	11.46	29.84	28.85	16.01	n.d.	4.94	n.d.	3.36
		9	6.10	12.91	42.02	21.13	4.69	3.52	5.40	n.d.	4.23
	17%	1	37.50	5.56	27.08	9.03	5.90	1.74	2.78	6.25	4.17
		2	35.50	5.76	29.08	9.53	5.30	1.76	2.63	6.03	4.41
		3	33.20	5.78	32.28	9.78	4.88	7.96	2.28	5.71	4.13
		4	36.85	6.42	35.83	10.85	5.41	2.18	2.53	6.34	4.58
		5	6.90	6.03	66.81	13.79	2.16	2.16	n.d.	n.d.	2.16
		6	13.83	9.47	32.52	20.63	14.32	n.d.	5.34	n.d.	3.88
		7	10.09	8.77	36.18	19.08	10.53	2.85	4.61	4.61	3.29
		8	12.63	8.00	36.84	22.74	9.05	n.d.	4.00	3.79	2.95
		9	10.94	9.11	40.10	20.57	7.55	3.13	4.95	n.d.	3.65

n.d. – not detected

Table S3. Technical data of Agrinose sensors

Type	Description	Detecting range (ppm)
TGS2600 - B00	General air contaminants, hydrogen and carbon monoxide	1 - 3 (H_2)
TGS2610 - C00	LP gas, butane	500 - 10 000
TGS2602 - B00	Ammonia, Hydrogen sulfide (high sensitivity to VOC and odorous gases)	1 - 30 (EtOH)
TGS2611 - C00	Natural gas, methane	500 - 10 000
TGS2603 - B01	Odors generated from spoiled foods	1 - 10 (EtOH)
TGS8100 MEMS	Cooking odors, gaseous air contaminants	1 – 30 H_2
TGS2620 - C00	Solvent vapours, volatile vapors, alcohol	50 - 5 000
AS – MLV - P2	CO, butane, methane, ethanol, hydrogen. Specifically designed for volatile organic compounds (VOCs)	10 - 10 000
